# Maternal and fetal outcomes of pregnant women with type 1 diabetes, a national population study

**DOI:** 10.18632/oncotarget.20952

**Published:** 2017-09-16

**Authors:** Shu-Fu Lin, Chang-Fu Kuo, Meng-Jiun Chiou, Shang-Hung Chang

**Affiliations:** ^1^ Division of Endocrinology and Metabolism, Department of Internal Medicine, Chang Gung Memorial Hospital, Chang Gung University, Taoyuan, Taiwan; ^2^ Division of Rheumatology, Allergy and Immunology, Department of Internal Medicine, Chang Gung Memorial Hospital, Taoyuan, Taiwan; ^3^ Office for Big Data Research, Chang Gung Memorial Hospital, Taoyuan, Taiwan; ^4^ Division of Cardiology, Department of Internal Medicine, Chang Gung Memorial Hospital, Taoyuan, Taiwan; ^5^ Chang Gung University, Taoyuan, Taiwan

**Keywords:** type 1 diabetes, pregnancy, outcomes

## Abstract

Pregnancy in women with type 1 diabetes is associated with poor maternal and neonatal outcomes. However, the risk of these outcomes has never been evaluated in an Asian national population. In this work, we report the maternal and fetal outcomes of pregnant women with type 1 diabetes in Taiwan. A total of 2,350,339 pregnancy records created between 2001 and 2012 were obtained from the National Health Insurance database and analyzed. Here, 630 pregnancy records were identified in women having type 1 diabetes. Compared with pregnant women without type 1 diabetes, pregnant women with the disease showed increased risk of multiple adverse outcomes, including preeclampsia, eclampsia, cesarean delivery, adult respiratory distress syndrome, pulmonary edema, sepsis, chorioamnionitis, pregnancy-related hypertension, puerperal cerebrovascular disorders, acute renal failure, and shock. Fetuses of type 1 diabetic mothers were at increased risk of stillbirth, premature birth, large for gestational age, low birth weight, and low Apgar score. Of the studied endpoints, only preeclampsia showed an improvement in the late period (2011–2012) when compared with the early period (2001–2010). These findings reveal that pregnant women with type 1 diabetes are at significantly increased risk of developing many adverse maternal and fetal outcomes. Therefore, pregnancy outcomes in women with type 1 diabetes should be improved.

## INTRODUCTION

The incidence of type 1 diabetes is about 0.1–36.5/100,000 persons with a 3.2%–5.3% annual increase rate in North America, Europe, and Asia [1]. The increasing number of patients with type 1 diabetes presents a significant public health challenge. The onset of type 1 diabetes usually occurs before the age of 30. Therefore, this disease could affect many women of reproductive age. Pregnancies in women with type 1 diabetes are often associated with increased risks of multiple maternal and fetal adverse outcomes. Improving care for diabetic patients to reduce a risk of associated complications is the goal of diabetes management [2].

The pregnancy outcomes in type 1 diabetic patients reported between 1991 and 2003 are grim [3, 4]. High rates of maternal and fetal adverse outcomes highlight the challenges physicians face in the management of pregnant patients with the disease. The causes of adverse pregnancy outcomes may include a high proportion of unplanned pregnancies with prior poor glycemic control, failure to maintain tight metabolic control during pregnancy, and preexisting medical comorbidities, such as nephropathy and hypertension [3–6].

Care for type 1 diabetes has advanced with time. Preconception and early pregnancy care to achieve optimal glycemic control has lowered perinatal mortality and the rate of fetal malformations [7]. The widespread adoption of blood glucose self-monitoring has enabled patients to adjust their insulin dose in a timely manner and modify their lifestyle to control their glucose levels. Compared with human insulin, a fast-acting insulin analogue has been demonstrated to lower the risk of fetal adverse outcomes [8]. New insulin delivery systems, including continuous subcutaneous insulin infusion, sensor-augmented pump therapy, and closed-loop insulin delivery, have also provided additional tools to improve glycemic control. In addition to glycemic control, multidisciplinary patient-centered care of type 1 diabetes before and during pregnancy likely has led to better gestational outcomes [9].

Despite these efforts, however, the potential impact of modern diabetes management on the outcomes of pregnant women with type 1 diabetes has not been evaluated in a large cohort. In this nationwide population-based study, we analyzed the Taiwan National Health Insurance Research Database, Birth Registry, and Death Registry, all of which are representative of the general population, to identify pregnancies between 2001 and 2012 and compare a comprehensive set of maternal and neonatal outcomes between the pregnancies of women with type 1 diabetes and those without the disease.

## RESULTS

### Maternal characteristics

We analyzed 2,350,339 pregnancies of ≥ 20 gestational weeks between 2001 and 2012 (Table 1). Type 1 diabetes was identified in 630 pregnancies. The mean ages of the mothers at the time of pregnancy in the reference and type 1 diabetes groups were 29.43 ± 4.87 years and 28.8 ± 4.96 years, respectively. The mean duration to have type 1 diabetes major illness card before pregnancy was 5.73 ± 3.93 years. The proportions of male infants were similar between the control and type 1 diabetes groups (52.1% and 52.22%, respectively). The control group presented a higher proportion of foreign nationality than the type 1 diabetes group (6.61% and 0.79%, respectively). Increasing numbers of pregnant women with type 1 diabetes were observed from 2001 to 2012. While a total of 18 pregnancies occurred in mothers with type 1 diabetes was observed in 2001, this number increased to 87 in 2012, a 4.8-fold increase. Compared with the control group, the type 1 diabetes group had a higher Charlson comorbidity index (0.18 and 0.03, respectively). Multiple comorbidities were more common in the pregnancies of type 1 diabetic women than in the pregnancies of the controls; these comorbidities included myocardial infarction (0.32% vs. 0%), congestive heart failure (0.79% vs. 0.06%), peripheral vascular disease (0.95% vs. 0.08%), cerebrovascular disease (0.32% vs. 0.07%), chronic pulmonary disease (1.75% vs. 0.61%), rheumatologic disease (1.27% vs. 0.68%), ulcer disease (5.87% vs. 0.97%), renal disease (1.75% vs. 0.04%), malignancy (0.32% vs. 0.08%), and mild liver disease (3.17% vs. 0.28%), demonstrating type 1 diabetic women had higher rates of a variety of disease before pregnancy compared with the control group.

**Table 1:** Baseline characteristics of pregnant women without type 1 diabetes and pregnant women with type 1 diabetes

Baseline characteristics	Without T1D (*n* = 2,349,709)	With T1D (*n*= 630)	*P* value
Age at pregnancy, mean (SD), y	29.43 (4.87)	28.80 (4.96)	**0.0011**
Age at pregnancy, No. (%), y			**0.0009**
< 25	425,202 (18.10)	150 (23.81)	
25–34	1,614,993 (68.73)	400 (63.49)	
> 34	309,514 (13.17)	80 (12.70)	
Male infant, No. (%)	1,224,250 (52.10)	329 (52.22)	**0.9519**
Nationality (foreigner), No. (%)	155,293 (6.61)	5 (0.79)	**< 0.0001**
Place of residence, No. (%)			**< 0.0001**
Urban	900,459 (38.32)	273 (43.33)	
Suburban	695,065 (29.58)	200 (31.75)	
Rural	640,048 (27.24)	150 (23.81)	
Unknown	114,137 (4.86)	7 (1.11)	
Income levels, No. (%)			**< 0.0001**
Quintile 1 (lowest)	708,404 (30.15)	178 (28.25)	
Quintile 2	180,351 (7.68)	88 (13.97)	
Quintile 3	279,514 (11.90)	79 (12.54)	
Quintile 4	376,502 (16.02)	94 (14.92)	
Quintile 5 (highest)	719,386 (30.62)	189 (30.00)	
Unknown	85,552 (3.64)	2 (0.32)	
Occupation, No. (%)			**< 0.0001**
Dependents of the insured individuals	702,759 (29.91)	178 (28.25)	
Civil servants, teachers, military personnel and veterans	35,028 (1.49)	6 (0.95)	
Non-manual workers and professionals	759,854 (32.34)	206 (32.70)	
Manual workers	420,652 (17.90)	111 (17.62)	
Other	345,864 (14.72)	127 (20.16)	
Unknown	85,552 (3.64)	2 (0.32)	
Year, No. (%)			**< 0.0001**
2001	216,981 (9.23)	18 (2.86)	
2002	212,471 (9.04)	28 (4.44)	
2003	199,539 (8.49)	40 (6.35)	
2004	202,613 (8.62)	34 (5.40)	
2005	195,274 (8.31)	51 (8.10)	
2006	194,317 (8.27)	64 (10.16)	
2007	192,538 (8.19)	52 (8.25)	
2008	186,467 (7.94)	56 (8.89)	
2009	182,721 (7.78)	60 (9.52)	
2010	157,711 (6.71)	61 (9.68)	
2011	188,230 (8.01)	79 (12.54)	
2012	220,847 (9.4)	87 (13.81)	
Charlson comorbidity index, mean (SD)†	0.03 (0.22)	0.18 (0.50)	**< 0.0001**
Charlson comorbidity index, No. (%)†			**< 0.0001**
0	2285,672 (97.27)	540 (85.71)	
1	56,578 (2.41)	68 (10.79)	
≥ 2	7,459 (0.32)	22 (3.49)	
Myocardial infarction	80 (0.00)	2 (0.32)	**0.0217**
Congestive heart failure	1,367 (0.06)	5 (0.79)	**< 0.0001**
Peripheral vascular disease	1,779 (0.08)	6 (0.95)	**< 0.0001**
Cerebrovascular disease	1,732 (0.07)	2 (0.32)	**0.0796**
Dementia	338 (0.01)	0 (0)	**NA**
Chronic pulmonary disease	14,426 (0.61)	11 (1.75)	**0.0021**
Rheumatologic disease	15,957 (0.68)	8 (1.27)	**0.0825**
Ulcer disease	22,720 (0.97)	37 (5.87)	**< 0.0001**
Hemiplegia or paraplegia	542 (0.02)	0 (0)	**NA**
Renal disease	919 (0.04)	11 (1.75)	**< 0.0001**
Any malignancy, including leukemia and lymphoma	1,803 (0.08)	2 (0.32)	**0.0853**
Mild liver disease	6,647 (0.28)	20 (3.17)	**< 0.0001**
Moderate or severe liver disease	176 (0.01)	0 (0)	**NA**
Metastatic solid tumor	377 (0.02)	0 (0)	**NA**
AIDS	174 (0.01)	0 (0)	**NA**

†Excluding diabetes mellitus in Charlson comorbidity index. T1D, type 1 diabetes; AIDS, acquired immunodeficiency syndrome; NA, not available.

### Maternal complications

Maternal outcomes in pregnant women with type 1 diabetes and those without the disease were evaluated in this study. No maternal mortality occurred within 30 days of delivery in 630 pregnancies with type 1 diabetes (Table 2). However, pregnant women with type 1 diabetes were usually at a much higher risk of developing adverse maternal events during their pregnancy than women without type 1 diabetes, even after adjusting for age and infant sex (Model 1) or age, infant sex, place of residence, income level, occupation, calendar year, and Charlson comorbidity index (Model 2). The risks of preeclampsia (adjusted odds ratio [OR], 10.27 [95% CI, 8.53–12.4]), eclampsia (adjusted OR, 15.6 [7.81–31.15]), and cesarean delivery (adjusted OR, 1.85 [1.72–2.0]) increased in the type 1 diabetes cohort. The risk of pulmonary diseases, including adult respiratory distress syndrome (adjusted OR, 38.34 [21.23–69.21]) and pulmonary edema (adjusted OR, 69.51 [32.81–147.25]), increased in pregnant women with type 1 diabetes. The risk of infection, including sepsis (adjusted OR, 11.04 [7.12–17.11]) and chorioamnionitis (adjusted OR, 2.99 [1.43–6.23]), also increased in pregnant women with type 1 diabetes. Pregnant women with type 1 diabetes were at a higher risk of developing pregnancy-related hypertension (adjusted OR, 8.34 [7.04–9.87]), puerperal cerebrovascular disorders (adjusted OR, 11.31 [5.13–24.94]), acute renal failure (adjusted OR, 46.55 [20.21–107.22]), shock (adjusted OR, 19.76 [8.64–45.18]), intracranial injuries (adjusted OR, 4.83 [2.54–9.18]), cardiac arrest/ventricular fibrillation (adjusted OR, 103.67 [30.83–3448.55]), acute myocardial infarction (adjusted OR, 30.85 [4.27–222.68]), severe anesthesia complications (adjusted OR, 12.07 [1.73–84.01]), and thrombotic embolism (adjusted OR, 30.98 [7.53–127.39]). These results demonstrate dismal outcomes in pregnant women with type 1 diabetes.

**Table 2:** Maternal outcomes among pregnant women without type 1 diabetes and pregnant women with type 1 diabetes

	No. of events (%)					
	Without T1D (*n* = 2,349,709)	With T1D (*n* = 630)	Crude OR (95% CI)	Model 1 OR (95% CI)^a^	Model 2 OR (95% CI)^b^	*P* value (Model 2)
Death ≤ 30 d postpartum	314 (0.01)	0 (0)	NA	NA	NA	
Preeclampsia	36,985 (1.57)	110 (17.46)	13.16 (10.57–16.39)	11.53 (9.57–13.90)	10.27 (8.53–12.4)	**< 0.0001**
Eclampsia	1,755 (0.07)	8 (1.27)	16.60 (7.95–34.68)	17.36 (8.71–34.59)	15.6 (7.81–31.15)	**< 0.0001**
Cesarean delivery	781,843 (33.27)	386 (61.27)	2.86 (2.38–3.43)	1.89 (1.76–2.04)	1.85 (1.72–2.0)	**< 0.0001**
Adult respiratory distress syndrome	999 (0.04)	14 (2.22)	53.48 (29.38–97.36)	53.99 (30.0–97.3)	38.34 (21.23–69.21)	**< 0.0001**
Pulmonary edema	325 (0.01)	7 (1.11)	81.52 (38.40–173.08)	83.44 (39.6–176)	69.51 (32.81–147.25)	**< 0.0001**
Sepsis	5,853 (0.25)	22 (3.49)	14.21 (8.89–22.69)	14.25 (9.13–22.25)	11.04 (7.12–17.11)	**< 0.0001**
Chorioamnionitis	8,057 (0.34)	7 (1.11)	3.27 (1.56–6.88)	3.28 (1.57–6.83)	2.99 (1.43–6.23)	**0.00345**
Pregnancy-related hypertension	53,917 (2.29)	132 (20.95)	11.16 (9.07–13.73)	9.51 (8.02–11.26)	8.34 (7.04–9.87)	**< 0.0001**
Puerperal cerebrovascular disorders	1,707 (0.07)	6 (0.95)	13.34 (5.97–29.79)	13.59 (6.13–30.1)	11.31 (5.13–24.94)	**< 0.0001**
Acute renal failure	503 (0.02)	7 (1.11)	52.22 (22.61–120.63)	53.79 (23.3–124)	46.55 (20.21–107.22)	**< 0.0001**
Shock	1,186 (0.05)	7 (1.11)	21.03 (8.68–50.99)	23.02 (10.01–52.93)	19.76 (8.64–45.18)	**< 0.0001**
Intracranial injuries	5,580 (0.24)	9 (1.43)	6.02 (3.12–11.60)	5.87 (3.09–11.1)	4.83 (2.54–9.18)	**< 0.0001**
Internal injuries of thorax, abdomen, and pelvis	966 (0.04)	2 (0.32)	3.74 (0.53–26.37)	3.78 (0.53–26.8)	3.13 (0.45–22.06)	**0.25209**
Cardiac arrest/ventricular fibrillation	112 (0.00)	4 (0.63)	133.44 (40.42–440.54)	140.49 (41.9–471)	103.67 (30.83–3448.55)	**0.00944**
Acute myocardial infarction	109 (0.00)	2 (0.32)	33.36 (4.66–238.63)	35.74 (5.01–255)	30.85 (4.27–222.68)	**0.00067**
Disseminated intravascular coagulation	1,541 (0.07)	2 (0.32)	2.56 (0.36–18.00)	2.57 (0.37–17.88)	2.31 (0.33–16.32)	**0.40129**
Severe anesthesia complications	300 (0.01)	2 (0.32)	12.70 (1.78–90.46)	12.62 (1.78–89.64)	12.07 (1.73–84.01)	**0.01187**
Thrombotic embolism	215 (0.01)	1 (1.16)	36.77 (9.13–148.19)	36.01 (8.96–144.71)	30.98 (7.53–127.39)	**< 0.0001**
Hysterectomy	1,861 (0.08)	1 (0.16)	1.91 (0.27–13.44)	2.19 (0.31–15.51)	2.28 (0.32–16.21)	**0.41019**
Antepartum hemorrhage	209,865 (8.93)	54 (8.57)	0.96 (0.72–1.29)	0.97 (0.74–1.26)	0.95 (0.72–1.23)	**0.69712**
Severe postpartum hemorrhage	63,345 (2.70)	25 (3.97)	1.47 (0.98–2.21)	1.46 (0.99–2.16)	1.33 (0.90–1.97)	**0.15480**

^a^Adjusted for age, infant sex. ^b^Adjusted for age, infant sex, place of residence, income, occupation, calendar year, and Charlson comorbidity index. T1D, type 1 diabetes; OR, odds ratio; CI, confidence interval; NA, not available.

### Fetal complications

The outcomes of fetuses in pregnant women with type 1 diabetes and those without type 1 diabetes were studied. Fetal outcomes were generally worse in the type 1 diabetes group than in the control group (Table 3). The fetuses of type 1 diabetic mothers showed higher risks of stillbirth (adjusted OR, 5.03 [3.45–7.34]), preterm birth (< 37 weeks; adjusted OR, 4.21 [3.78–4.71]), large for gestational age (adjusted OR, 4.44 [3.99–4.95]), low birth weight (< 2,500 g; adjusted OR, 2.38 [1.99–2.84]), and a low Apgar score (< 7 at 5 minutes; adjusted OR, 4.98 [3.05–8.15]). However, the fetuses of type 1 diabetic mothers showed a lower risk of being small for their gestational age (adjusted OR, 0.62 [0.45–0.85]). The incidence of fetal abnormalities was not significantly increased in the type 1 diabetes group (adjusted OR, 1.14 [0.84–1.56]) compared with that in the control group.

**Table 3:** Fetal outcomes among pregnant women without type 1 diabetes and pregnant women with type 1 diabetes

	No. of events (%)					
	Without T1D (*n* = 2,349,709)	With T1D (*n* = 630)	Crude OR (95% CI)	Model 1 OR (95% CI)^a^	Model 2 OR (95% CI)^b^	*P* value (Model 2)
Stillbirth	17,338 (0.74)	25 (3.97)	5.40 (3.62–8.07)	5.53 (3.80–8.06)	5.03 (3.45–7.34)	**< 0.0001**
Preterm birth (< 37 week)	178,062 (7.58)	216 (34.29)	2.96 (2.37–3.69)	4.58 (4.09–5.13)	4.21 (3.78–4.71)	**< 0.0001**
Large for gestational age	231,036 (9.83)	257 (40.79)	5.87 (4.95–6.97)	4.27 (3.84–4.75)	4.44 (3.99–4.95)	**< 0.0001**
Small for gestational age	228,163 (9.71)	42 (6.67)	0.67 (0.48–0.94)	0.67 (0.49–0.92)	0.62 (0.45–0.85)	**0.00298**
Low birth weight (< 2500 g)	151,112 (6.43)	109 (17.30)	6.23 (5.25–7.40)	2.68 (2.24–3.22)	2.38 (1.99–2.84)	**< 0.0001**
Apgar score < 7 at 5 min	12,198 (0.52)	15 (2.38)	4.85 (2.90–8.11)	4.79 (2.91–7.89)	4.98 (3.05–8.15)	**< 0.0001**
Fetal abnormalities, any	105,833 (4.50)	37 (5.87)	1.30 (0.94–1.81)	1.33 (0.98–1.81)	1.14 (0.84–1.56)	0.41291

^a^Adjusted for age, infant sex. ^b^Adjusted for age, infant sex, place of residence, income, occupation, calendar year, and Charlson comorbidity index. T1D, type 1 diabetes; OR, odds ratio; CI, confidence interval.

### Temporal trend of pregnancy outcomes

**Figure 1:**
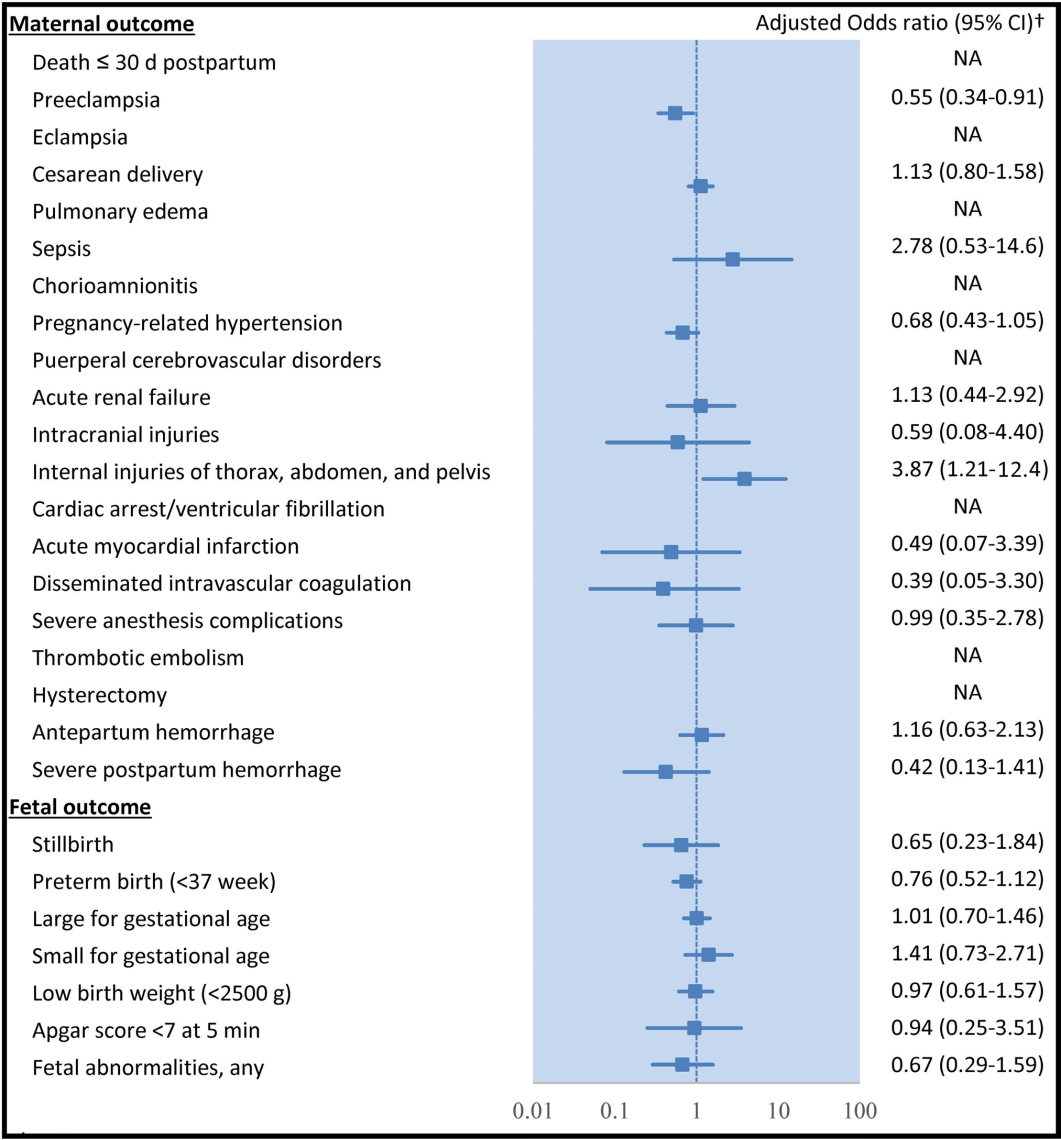
Comparison of outcomes between early period (2001–2010) and late period (2011–2012) among pregnant women with type 1 diabetes Type 1 diabetic women had a lower risk of developing preeclampsia during the late period than the early period. The differences in other pregnancy outcomes did not show any statistical significance between the two periods. † Adjusted for age, infant sex, place of residence, income, occupation, calendar year, and Charlson comorbidity index. CI, confidence interval; NA, not available.

The dataset including a relative large sample size of pregnant women with type 1 diabetes over a long time span allowed us to evaluate the potential changes in pregnancy outcomes over time (Figure 1). Since June 1, 2010, 120 blood glucose test strips per month were reimbursed by the National Health Insurance Administration for patients with type 1 diabetes. This major favorable policy may impact the care and outcomes in pregnant women with type 1 diabetes in Taiwan. Therefore, we assessed the pregnancy outcomes of women with type 1 diabetes between two periods: 2001–2010 (early period) and 2011–2012 (late period). In addition, this comparison allowed us to identify potential changes in pregnancy outcomes in the most recent 2 years. The results demonstrated that type 1 diabetic women generally presented a lower risk of developing preeclampsia (adjusted OR, 0.55 [0.34–0.91]) during the late period than during the early period. Nevertheless, differences in other maternal and fetal outcomes in pregnant women with type 1 diabetes did not show any statistical significance between the two periods.

## DISCUSSION

This population-based cohort study reports two major findings: 1) The risk of complications associated with type 1 diabetes during pregnancy is high and 2) the risk of preeclampsia recently decreased in pregnant women with type 1 diabetes.

In this study, we found a 4.8-fold increase in the number of pregnant women with type 1 diabetes between 2001 and 2012. This observation highlights the importance of improvements in the gestational outcomes of this population. However, the incidence of type 1 diabetes had not changed significantly (around 2.75–3.56 per 100,000 persons) between 2000 and 2009 in Taiwan [10].

This study showed strong correlations between type 1 diabetes and multiple adverse outcomes, including pregnancy-related hypertension, preeclampsia, eclampsia, cesarean delivery, stillbirth, and preterm birth. In addition to these well-recognized adverse outcomes, we also found that patients with type 1 diabetes were associated with high risks of other major morbidities, including adult respiratory distress syndrome, pulmonary edema, sepsis, chorioamnionitis, puerperal cerebrovascular disorders, acute renal failure, and shock. These data emphasize that type 1 diabetes is associated with increased adverse outcomes in both mothers and fetuses.

Our data revealed alarmingly higher risks in many adverse outcomes in pregnant women with type 1 diabetes as compared with those without the disease. These findings were unexpected. The National Health Insurance program was built in 1995 in Taiwan to reduce barriers to the health care system for all citizens [11]; nearly all diabetes-related treatment costs are reimbursed for type 1 diabetes, and diabetes care is provided by teams consisting of physicians, certified educators, and dietitians. However, these management measures appeared to have been insufficient to lead to satisfactory results in pregnant women with type 1 diabetes between 2001 and 2012 in the country.

We found that the only improvement in outcome was a lower risk of preeclampsia in the late period as compared with that in the early period in pregnant women with type 1 diabetes. The reasons behind for this improvement are unclear. Better glucose and blood pressure control, dietary and lifestyle modification, low-dose aspirin, and calcium supplementation in women with low dietary calcium intake have been reported to reduce the risk of preeclampsia [12–16]. We further supposed that the availability of free glucose test strips had relieved the financial stress associated with the disease and led to better diabetes care and outcomes. The decreased risk of preeclampsia is of high clinical significance because preeclampsia is associated with maternal morbidity and mortality during pregnancy and is a strong predictor of future cardiovascular disease in mothers [17, 18].

Except preeclampsia, no improvement in maternal and fetal outcomes in the late period was found among pregnant women with type 1 diabetes. Similar to our results, a recent study also reported difficulty in improving pregnancy outcomes in women with type 1 diabetes [19]; high rates of adverse pregnancy events, including preeclampsia, cesarean delivery, premature birth, and large for gestational age, were reported in that study population. The excellent glycemic control achieved by the sensor-augmented pump therapy and closed-loop insulin delivery system described in that study was worthy of note. These data indicate that optimal glycemic control during pregnancy is insufficient to achieve promising pregnancy outcomes in this population.

To obtain greater insights into the trend of pregnant outcomes in women with type 1 diabetes from 2001 to 2012, we perform a joinpoint regression analysis (Supplementary Figure 1). Among the nine outcomes including over 36 events during the 12-year study period, preeclampsia demonstrated the greatest improvement (−4.6% of average annual change), followed by pregnancy-related hypertension (−3.6%); however, none of these outcomes reached statistical significance according to this analysis.

This study showed a higher prevalence of multiple comorbidities in women with type 1 diabetes than in women without the disease. These comorbidities include myocardial infarction, congestive heart failure, renal disease, chronic pulmonary disease, liver disease, and peripheral vascular disease. Similar to our results, a recent report noted the frequent prevalence of diabetes-related complications and comorbidities among young adults who had been diagnosed with type 1 diabetes [20]. Early monitoring of the youth with diabetes for development of complications is suggested.

Our data revealed a decreased risk of premature birth in pregnant women with type 1 diabetes in the late period as compared with that in the early period, although the difference was not statistically significant. Preeclampsia is a major cause of iatrogenic premature birth because delivery remains the treatment of last resort for preeclampsia [21]. Thus, decreasing the risk of preeclampsia could reasonably be supposed to lead to an improvement in the rate of premature birth.

Several strengths are highlighted by this study. First, this study analyzed a large population-based National Health Insurance Database in Taiwan. The national health insurance has provided universal coverage of medical care to around 99.0%–99.5% of the entire population (approximately 23 million persons) since 1995. The National Health Insurance Research Database was established to facilitate research on health care in 1997. This database includes information on personal features (sex, date of birth, place of residence, income level, and employment); details of clinical information, including diagnoses related to inpatient and outpatient care and procedures; and prescribed medications and operations. Second, this study enrolled 2,350,339 pregnancies, including 630 pregnancies with type 1 diabetes; thereby providing some statistical power to detect differences in multiple pregnant outcomes between two cohorts.

This study also presents a number of limitations. First, the dataset lacked information regarding physical activity, smoking habits, alcohol consumption, family history, and use of over-the-counter drugs. These confounding factors are likely to impact maternal and fetal outcomes. Second, we did not include pregnancies < 20 weeks. Therefore, the impacts of type 1 diabetes on early pregnancy must be clarified. Third, our results represent the gestational outcomes of women with type 1 diabetes only in Taiwan. However, two studies conducted in the United Kingdom and France demonstrated similar results, thus suggesting that the pregnancy outcomes of women with type 1 diabetes remain unfavorable in some countries [19, 22]. Fourth, we did not assess the effects of treatment drugs (human insulin, insulin analogues) on the outcomes of pregnant women with type 1 diabetes. Fifth, because the numbers of most maternal adverse event were limited (< 10 events) in pregnant women with type 1 diabetes, we did not perform logistic regression stratified for each covariate, such as age, place of residence, income level, and occupation.

In conclusion, our data indicate that type 1 diabetes remains a significant disease threatening pregnant women and their offspring. Clinicians should be aware of this clinical situation.

## MATERIALS AND METHODS

### Study participants

This study used data from the Taiwan National Health Insurance Research Database established by the Bureau of National Health Insurance [11, 23]. Pregnancies of ≥ 20 weeks occurring in Taiwan between January 1, 2001 and December 31, 2012 were identified from the database, where clinical information regarding pregnant women had been recorded. In addition, gestational age, birth weight, Apgar score, and fetal outcomes were obtained from the birth registry recorded by obstetricians. The reporting of fetal information to the Ministry of Health and Welfare is mandated by law. This study was approved by the Institutional Review Board of Chang Gung Memorial Hospital and the Ministry of Health and Welfare, Taiwan (104–8042B). The need for informed consent was waived because all data were completely anonymous.

### Ascertainment of type 1 diabetes

Subjects who had a type 1 diabetes major illness card recorded in the Catastrophic Illness Patient Database were considered to have type 1 diabetes in this study. The type 1 diabetes major illness card is issued after a medical claim review and approval by experts of the national health insurance administration. The clinical and laboratory characteristics indicating type 1 diabetes include 1) acute symptoms of diabetes, 2) presence of diabetic ketoacidosis, and 3) presence of autoimmune markers (including autoantibodies to insulin, islet cell, glutamic acid decarboxylase 65 and tyrosine phosphatase-related islet antigen 2) [24, 25].

### Study outcomes

Maternal adverse outcomes were selected according to the Centers for Disease Control and Prevention's severe maternal morbidity composite outcomes [26]. These measurements are frequently used to indicate outcomes related to hospitalization during pregnancy, including death within 30 days of delivery, cesarean delivery, and pregnancy-related complications, such as pregnancy-related hypertension, preeclampsia, eclampsia, hemorrhage, preterm labor, and chorioamnionitis [27].

Fetal adverse outcomes included poor or excessive fetal growth, fetal abnormalities, low Apgar score, and stillbirth. Detailed codes of the International Classification of Diseases, 9th Revision (ICD-9), codes of the Diagnosis-Related Group (DRG), and items of the Birth Registry used in this study are listed in Supplementary Table 1.

### Statistical analysis

The effects of type 1 diabetes on maternal and fetal adverse outcomes were analyzed. The covariates for multivariate analysis included age and infant sex for Model 1 and age, infant sex, place of residence, income level, occupation, calendar year, and Charlson comorbidity index for Model 2. ORs with 95% confidence intervals were calculated using logistic regression for each maternal and fetal outcome between the type 1 diabetes and control groups, using SAS, version 9.4 (SAS Institute Inc.). This analysis was also preformed to evaluate the ORs of outcomes in pregnant women with type 1 diabetes between the early period (2001–2010) and the late period (2011–2012). Similar analyses were used to evaluate pregnant complications in prior reports [4, 18, 22, 28]. *P* < 0.05 was considered statistically significant.

## SUPPLEMENTARY MATERIALS FIGURES AND TABLES




