# Supplementary material for: Maternal and fetal outcomes of pregnant women with type 1 diabetes, a national population study
Source: Oncotarget. 2017 Sep 16;8(46):80679–87. doi: 10.18632/oncotarget.20952 (PMC5655230; doi:10.18632/oncotarget.20952)
Supplement: Supplementary file 1 [file oncotarget-08-80679-s001.pdf]

Maternal and fetal outcomes of pregnant women with type 1 diabetes, a national population study

SUPPLEMENTARY MATERIALS

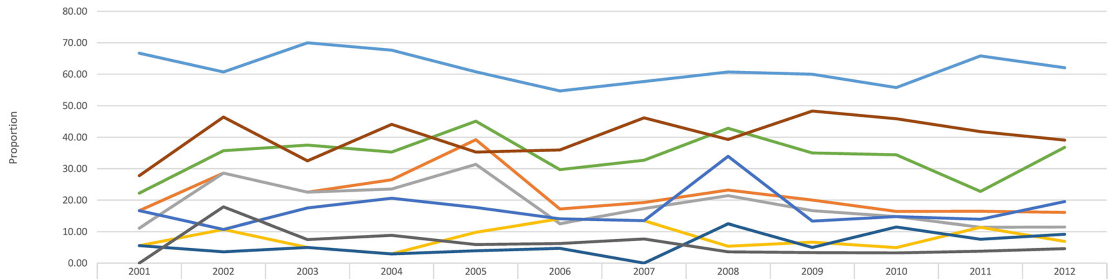

Calendar year	2001	2002	2003	2004	2005	2006	2007	2008	2009	2010	2011	2012
Cesarean delivery (AAPC= -0.8 (-2.2 to 0.6))	66.67	60.71	70.00	67.65	60.78	54.69	57.69	60.71	60.00	55.74	65.82	62.07
Pregnancy-related hypertension (AAPC=-3.6 (-8.1 to 1.1))	16.67	28.57	22.50	26.47	39.22	17.19	19.23	23.21	20.00	16.39	16.46	16.09
Preeclampsia (AAPC=-4.6 (-10.5 to 1.7))	11.11	28.57	22.50	23.53	31.37	12.50	17.31	21.43	16.67	14.75	11.39	11.49
Antepartum hemorrhage (AAPC=1.8 (-7.3 to 11.7))	5.56	10.71	5.00	2.94	9.80	14.06	13.46	5.36	6.67	4.92	11.39	6.90
Low birth weight (AAPC= 0.9 (-4.7 to 6.8))	16.67	10.71	17.50	20.59	17.65	14.06	13.46	33.93	13.33	14.75	13.92	19.54
Preterm birth (AAPC = 0.3 (-3.9 to 4.6))	22.22	35.71	37.50	35.29	45.10	29.69	32.69	42.86	35.00	34.43	22.78	36.78
Small for gestational age (AAPC=N/A)	5.56	3.57	5.00	2.94	3.92	4.69	0.00	12.50	5.00	11.48	7.59	9.20
Large for gestational age (AAPC=2.2 (-0.7 to 5.2))	27.78	46.43	32.50	44.12	35.29	35.94	46.15	39.29	48.33	45.90	41.77	39.08
Fetal abnormalities, any (AAPC=N/A)	0.00	17.86	7.50	8.82	5.88	6.25	7.69	3.57	3.33	3.28	3.80	4.60

Supplementary Figure 1: The trend of pregnancy outcomes in women with type 1 diabetes from 2001 to 2012. The changes of outcome rate in pregnant women with type 1 diabetes were evaluated using Jointpoint regression analysis. Results are proportions of event and annual average percentage change (AAPC) with 95% confidence interval.

Supplementary Table 1: International Classification of Diseases, ninth revision (ICD-9) codes, Diagnosis-Related Group (DRG) codes, and items of birth registry used in this study. See Supplementary_Table_1
