# Supplementary material for: Maternal and fetal outcomes of pregnant women with type 1 diabetes, a national population study
Source: Oncotarget. 2017 Sep 16;8(46):80679–87. doi: 10.18632/oncotarget.20952 (PMC5655230; doi:10.18632/oncotarget.20952)
Supplement: Supplementary file 2 [file oncotarget-08-80679-s002.docx]

Supplementary Table 1: International Classification of Diseases, ninth revision (ICD-9) codes, Diagnosis-Related Group (DRG) codes, and items of birth registry used in this study

| Disease | ICD-9 Diagnosis Codes | ICD-9 Procedure Codes | DRG code | Other |
| --- | --- | --- | --- | --- |
| Delivery | V27, 650, 651, 652 | 72, 73, 74 | 0371A, 0373B, 0373C |  |
| Cardiovascular |  |  |  |  |
| Acute myocardial infarction | 410.xx |  |  |  |
| Congestive heart failure | 402.01, 402.11, 402.91, 425.x, 428.x, 429.3, 404.01, 404.03, 404.11, 404.13, 404.91, 404.93 |  |  |  |
| Peripheral vascular disease | 440.x, 441.x, 442.x, 443.1-443.9, 447.1, 785.4 | 38.13-38.14, 38.16, 38.18, 38.33-38.34, 38.36, 38.38, 38.43-38.44, 38.46, 38.48, 39.22-39.26, 39.29 |  |  |
| Cerebrovascular disease | 362.34, 430-436, 437-437.1, 437.9, 438, 781.4, 784.3,997.0 | 38.12, 38.42 |  |  |
| Cardiac arrest/ventricular fibrillation | 427.41, 427.42, 427.5 |  |  |  |
| Shock | 669.1x, 785.5x |  |  |  |
| Pregnancy-related hypertension | 642.3x, 642.4x, 642.5x, 642.6x, 642.7x, 642.9x, |  |  |  |
| Puerperal cerebrovascular disorders | 430, 431, 432.x, 433.xx, 434.xx, 436, 437.x, 671.5x, 674.0x, 997.2, 999.2 |  |  |  |
| Thrombotic embolism | 415.1x, 673.0x, 673.2x, 673.3x, 673.8x, |  |  |  |
| Complication during delivery |  |  |  |  |
| Antepartum hemorrhage | 641.1x, 641.2x, 641.3x, 641.8x, 641.9x |  |  |  |
| Severe postpartum hemorrhage | [666.0x-666.3x] plus either blood transfusion [99.0x] or hysterectomy [68.3x-68.9] |  |  |  |
| Chorioamnionitis | 658.4x |  |  |  |
| Cesarean delivery |  |  |  | Birth Registry |
| Surgical complications |  |  |  |  |
| Severe anesthesia complications | 668.0x, 668.1x, 668.2x |  |  |  |
| Thorax, abdomen, and pelvis injuries | 860.xx-869.xx |  |  |  |
| Intracranial injuries | 800.xx, 801.xx, 803.xx, 804.xx, 851.xx, 854.xx |  |  |  |
| Hysterectomy | 68.3x-68.9 |  |  |  |
| Other |  |  |  |  |
| Dementia | 290.x, 331-331.2 |  |  |  |
| Chronic pulmonary disease | 415.0, 416.8-416.9, 491.x-494, 496 |  |  |  |
| Rheumatologic disease | 710.x, 714.x |  |  |  |
| Ulcer disease | 531.xx-534.xx |  |  |  |
| Hemiplegia or paraplegia | 342.x, 344.x |  |  |  |
| Renal disease | 585-586, V42.0, V45.1, V56.x | 39.27, 39.42, 39.93-39.95, 54.98 |  |  |
| Any malignancy, including leukemia and lymphoma | 140.x-171.x, 174.x-195.x, 200.xx-208.x, 273.0, 273.3, V10.46 |  |  |  |
| Mild liver disease | 571.2, 571.5-571.6, 571.8-571. 9 |  |  |  |
| Moderate or severe liver disease | 572.2-572.4, 456.0-456.2x | 39.1, 42.91 |  |  |
| Metastatic solid tumor | 196.x-199.x |  |  |  |
| Acquired immunodeficiency syndrome | 042.x-044.x |  |  |  |
| Acute renal failure | 584.x, 669.3x |  |  |  |
| Adult respiratory distress syndrome | 518.5, 518.81, 518.82, 518.84, 799.1 |  |  |  |
| Sepsis | 038.xx, 995.91, 995.92 |  |  |  |
| Disseminated intravascular coagulation | 286.6, 286.9, 666.3x |  |  |  |
| Preeclampsia | 642.4x, 642.5x, 642.6x, 642.7x |  |  |  |
| Eclampsia | 642.6x |  |  |  |
| Pulmonary edema | 428.1, 518.4 |  |  |  |
| Low birth weight, <2500 g |  |  |  | Birth Registry |
| Preterm birth, <37 week |  |  |  | Birth Registry |
| Apgar score <7 at 5 minutes |  |  |  | Birth Registry |
| Fetal abnormalities, any | 655.xx |  |  | Birth Registry |
| Stillbirth |  |  |  | Birth Registry |
